# Repeal of the Medical Law of Ohio

**Published:** 1833

**Authors:** 


### Art. XIII. Repeal of the Medical Law of Ohio. Qualified Approval. Suggestions about another Law.

Our last Legislature repealed the law regulating the Practice of Medicine, a fact we forgot to mention to our distant readers, in either of the preceding numbers of this volume. This was an ancient law of our State. Twenty two years ago, we recollect to have made a winter visit to Chillicothe, for the purpose of attending the first convention under that law. Dr. Canby, of Lebanon, and Dr. Parsons, of Columbus, also attended, and Dr. Scott and Dr. Edmiston, of Chillicothe, were delegates; but five could not form a quorum, and no convention was organized. From that time forward to last winter, Ohio had, nearly or all the time, a law constituting the physicians of the State into district societies, authorising general societies, and denouncing quackery under heavy penalties. But during this period, it took deeper root in our community than any other in the Union; and has even established a college and journal of its own! A memorable example of the inefficiency of Legislation, when *popular* sentiment does not co-operate. We have no doubt, that the laws of Ohio have contributed to rear the seat, on which empyricism now sits enthroned. The people have never looked to the execution of the Medical law, and when a society has at any time endeavored to enforce it, they have taken sides with the *oppressed!* A perception of the total inefficiency of the law, led the General Medical Society, at its last meeting, to petition the Legislature for its repeal; the prayer was granted, and the corporation making it dissolved. We cannot help thinking, however, that the members of that respectable body, might have devised some intermediate course, that would have been preferable, not as to the suppression of quackery, but the improvement of the profession. They could have recommended the abolition of licenses, penalties and disabilities, but retained the organization into districts and a General o r State society, and thus have made it a means of promoting scientific and professional improvement. It was the prevailing neglect of the means of such improvement, that rendered us all indifferent to the continuance of the law.

Having never ourselves been other than an honoray member of the General society, we have had no opportunity, personally, of urging upon that body, to occupy itself in its annual meetings, on subjects of scientific research; but on the approach of its last meeting, we ventured to address to it a memorial, suggesting several subjects for its consideration; before, however, it reached the Capital, the members had dispersed, and soon afterwards, the law under which they assembled, was repealed. Believing that some of the suggestions contained in that communication, are worthy fixing the attention of our brethren throughout the State, we shall briefly set forth a few of them, in the manner in which they now present themselves:

1. Had the District Societies been preserved, or rather were they to be revived, (not to regulate the practice of physic, but to promote scientific research,) every one would contain a body of inquirers, who would aid and animate each other, in various useful labors. It would be but a few hours' ride from the most remote point of a District, to the place of meeting, and thus frequent conventions of its intelligent, enterprising and zealous physicians, might be held. At these meetings, they might report and compare their modes of practice in the current and the endemic diseases of the District, and thus reciprocally improve each other's practice.

2. They might appoint a meteorologist for the District, and, supplying him with a thermometer, barometer, hygrometer, electrometer, pluviameter, vane, and other instruments for estimating atmospheric phenomena, enable him to keep, and report at stated periods, to the society, a valuable climatorial register.

3. Each society might form an *herbarium*, of the plants within its limits, which would inspire and gratify a taste for Botany among its pupils; while experiments might be instituted on those which are curative, and new medicinal virtues ascertained.

4. Every society might form a cabinet of the minerals within its District; noting their localities, and the relations they bear to each other.

5. A library of current and periodical works might, at a small expense, be established, which would put all the members in possession of the improvements that are daily made in the profession elsewhere; and which might, for a small compensation, be made accessible to the poor students of the District, many of whom prosecute their studies in books the most inappropriate.

6. The different societies might reciprocally exchange specimens of plants and minerals, until each would acquire a part of what had been collected by every other.

7. Once in two or three years, each District might elect two or more of its distinguished members, as delegates to a State society, to meet annually in *Columbus*, at a pleasant and healthy season. This society should continue in session for a number of days, and consider itself to be a learned association, convened for important purposes. To this society, the Districts should send copies of their meteorological registers; topographical, mineralogical, and geological descriptions of their Districts; reports of new and difficult forms of disease; an account of new remedies; and all such other observations, discoveries and inventions, in medicine and the collateral sciences, as might be regarded of interest to the profession. From the mass of communications thus made, the General Society might select such as seemed worthy of preservation, and arrange them into a volume of transactions. Such a volume might be published annually, differing in size according to the varying number of valuable communications before the society. Every physician of the State, not to look further, would undoubtedly purchase a copy of it, and that would be sufficient to defray the expense of publishing.

From this simple method, many important advantages would result.—A laudable emulation would be excited among the physicians of each District, and the different Districts would soon come to vie with each other, in labors for the advancement of the profession; the advantages of students of medicine, while in their private pupilage, would be greatly improved; and materials for an accurate history of the diseases, climate, medical plants, medicinal springs, and useful minerals, would be rapidly accumulated,—results of the deepest interest to the Profession, and the State at large; and which, without an associated effort, are not like to be attained in a century.

Many subordinate benefits, it may be conceived, would, from time to time, be the natural consequences of the action here recommended. We shall refer to one only. The place of meeting of each society, might be made a place of deposit for *Vaccine infection;* and thus the most remote and insulated members of the profession be supplied and incited to its employment; while occasional publications, by the Districts, or the General Society, addressed to the people at large, might rouse them from their lethargy, and speedily diminish the number, amounting no doubt to hundreds of thousands, who are now liable to be destroyed by the small-pox.

Under these various impressions, we beg leave to recall the attention of the *Profession* and the *Legislature*, to the subject of a Medical Law, which, while it should confer no power on the incorporated body, to regulate the practice of medicine, or establish fees, should embrace all the graduates and licentiates of the State, and all the members of the late District Societies, who might still be alive. The right should not be granted to the Districts, of imposing fines upon their members for non-attendance, nor of *collecting* taxes from them by judicial process; but each corporation should be at liberty to exclude from the use of its library, cabinet, herbarium, and vaccine deposit, all members who might neglect to aid in their establishment and support.

